# The role of psychological attribution in responses to weight stigma

**DOI:** 10.1002/osp4.437

**Published:** 2020-07-11

**Authors:** Mary A. Gerend, Angelina R. Sutin, Antonio Terracciano, Jon K. Maner

**Affiliations:** ^1^ Department of Behavioral Sciences and Social Medicine, College of Medicine Florida State University Tallahassee Florida USA; ^2^ Department of Geriatrics, College of Medicine Florida State University Tallahassee Florida USA; ^3^ Department of Psychology, College of Arts and Sciences Florida State University Tallahassee Florida USA

**Keywords:** Perceived weight discrimination, stigma, stress

## Abstract

**Objective:**

Weight discrimination is associated with numerous negative health consequences. Little is known about early‐stage psychological mechanisms that explain variability in responses to weight discrimination among people with obesity. This study tested the hypothesis that attributing negative social evaluation to one's weight would be associated with stigma‐related stress responses (eg, reduced cognitive functioning and self‐esteem, increased negative affect and cortisol), especially among people who had experienced frequent weight discrimination in the past.

**Methods:**

Adults (N = 109) with obesity were randomly assigned to receive a mildly positive (control) versus negative social evaluation. The extent to which participants attributed the negative evaluation to their physical appearance was assessed, along with negative affect, social and appearance self‐esteem, cognitive functioning and salivary cortisol.

**Results:**

Participants who had experienced frequent weight discrimination in the past were more likely to attribute the negative evaluation to their appearance. Participants who attributed the negative evaluation to their appearance in turn experienced elevated negative affect, lower appearance self‐esteem and worse cognitive functioning.

**Conclusions:**

This study is among the first to identify attribution as an early‐stage process underlying responses to weight stigma. Attribution may be a key psychological factor conferring risk for or protection from the negative effects of weight stigma.

## 1 INTRODUCTION

Weight stigma – the social devaluation of people with obesity as expressed through stereotypes, prejudice and discrimination – is highly prevalent in the United States.^1^ As many as 42% of adults report experiencing weight‐based discrimination, with higher rates typically observed among women and people with higher body weight (eg, body mass index [BMI] > 35 kg/m^2^).^2^, ^3^, ^4^ Weight discrimination occurs frequently in interpersonal relationships and is common across employment, education and healthcare settings.^1^, ^5^, ^6^ Weight discrimination can range from minor forms of differential treatment such as receiving a disparaging look or an insensitive comment to major acts such as being denied a job promotion because of one's weight.^5^, ^7^ Over time, repeated experiences with weight discrimination, coupled with an awareness of negative stereotypes and the socially devalued nature of people with obesity, can lead some individuals to internalize weight bias (ie, engage in self‐devaluation and chronically fear being stigmatized for their weight).^8^, ^9^, ^10^

Being the target of weight discrimination is associated with a range of negative consequences for psychological and physical health.^9^, ^11^, ^12^ Perceived weight discrimination is correlated with higher risk of low self‐esteem, poor body image and depression,^11^ as well as worse self‐reported health,^13^ poorer diabetes management,^14^ increased risk of dementia^15^, ^16^ and increased mortality risk.^17^ Moreover, perceived weight discrimination is associated with increased risk for the development and maintenance of obesity.^18^, ^19^ For instance, a study by Sutin and colleagues^19^ found that adults without obesity at baseline who reported experiencing unfair treatment due to their weight were 2.5 times more likely to meet BMI criteria for obesity 4 years later, even while controlling for baseline BMI. Indeed, in some cases, weight discrimination is a stronger predictor of negative health outcomes than is higher BMI itself.^17^, ^20^ Weight discrimination is believed to contribute to poor health and obesity maintenance via cognitive (eg, impaired self‐regulation and executive functioning), emotional (eg, psychological distress), physiological (eg, secretion of cortisol as triggered by activation of the hypothalamic‐pituitary‐adrenal axis) and behavioural (eg, increased eating) pathways that are activated in response to the psychological stress of being stigmatized.^9^, ^13^, ^21^, ^22^, ^23^, ^24^, ^25^, ^26^, ^27^, ^28^

Compared with the long‐term health consequences of weight stigma,^9^, ^11^, ^12^ less is known about early‐stage psychological mechanisms that underlie people's reactions to weight discrimination. The current study investigated whether people's responses to weight stigma are linked with biased patterns of psychological attribution. Attribution refers to the processes through which people generate inferences about the intentions and motivations (among other factors) that guide people's behaviour.^29^, ^30^ Attributions reflect the operation of cognitive schemas that assist people in interpreting and assigning meaning to social interactions.^29^, ^30^, ^31^, ^32^ Indeed, attributions play a key role in shaping people's interpretations of the social environment.^29^, ^30^

Attributions may be especially important when the motives guiding someone's behaviour are not immediately apparent, as is sometimes the case when people experience weight‐based discrimination. Although weight stigma remains a socially acceptable form of bias^1^ that often involves overt references to weight (eg, a celebrity with obesity is explicitly derogated for their weight on social media), many instances of weight stigma are less explicit, and thus, the motives underlying the unfair treatment are unknown. Supporting this notion, qualitative research on people's experiences with obesity documents instances in which participants suspected they were mistreated for their weight although weight was never explicitly mentioned.^7^ To illustrate, imagine a situation in which a group of people at a party act disparagingly toward a female partygoer with obesity but stop short of saying anything about her weight. In this example, the woman's interpretation of the situation and, in particular, whether she attributes the group's disparaging behaviour to her weight, will likely affect her responses to their behaviour. In sum, when instances of weight stigma are unclear or ambiguous, attributional processes may play an especially important role in shaping people's interpretations and responses.

Theories of attribution^33^ and research on cognitive appraisals and stress^34^, ^35^ suggest that people experience heightened stress responses when negative experiences are attributed to internal (vs. external) and controllable (vs. uncontrollable) causes. Obesity is a highly visible, socially devalued trait that is stereotypically viewed as controllable and due to internal causes, and thus, people with obesity are often presumed to be personally responsible for their weight.^5^ For these reasons, attributing a negative social encounter to one's body weight should elicit high levels of psychological stress.

**FIGURE 1:**
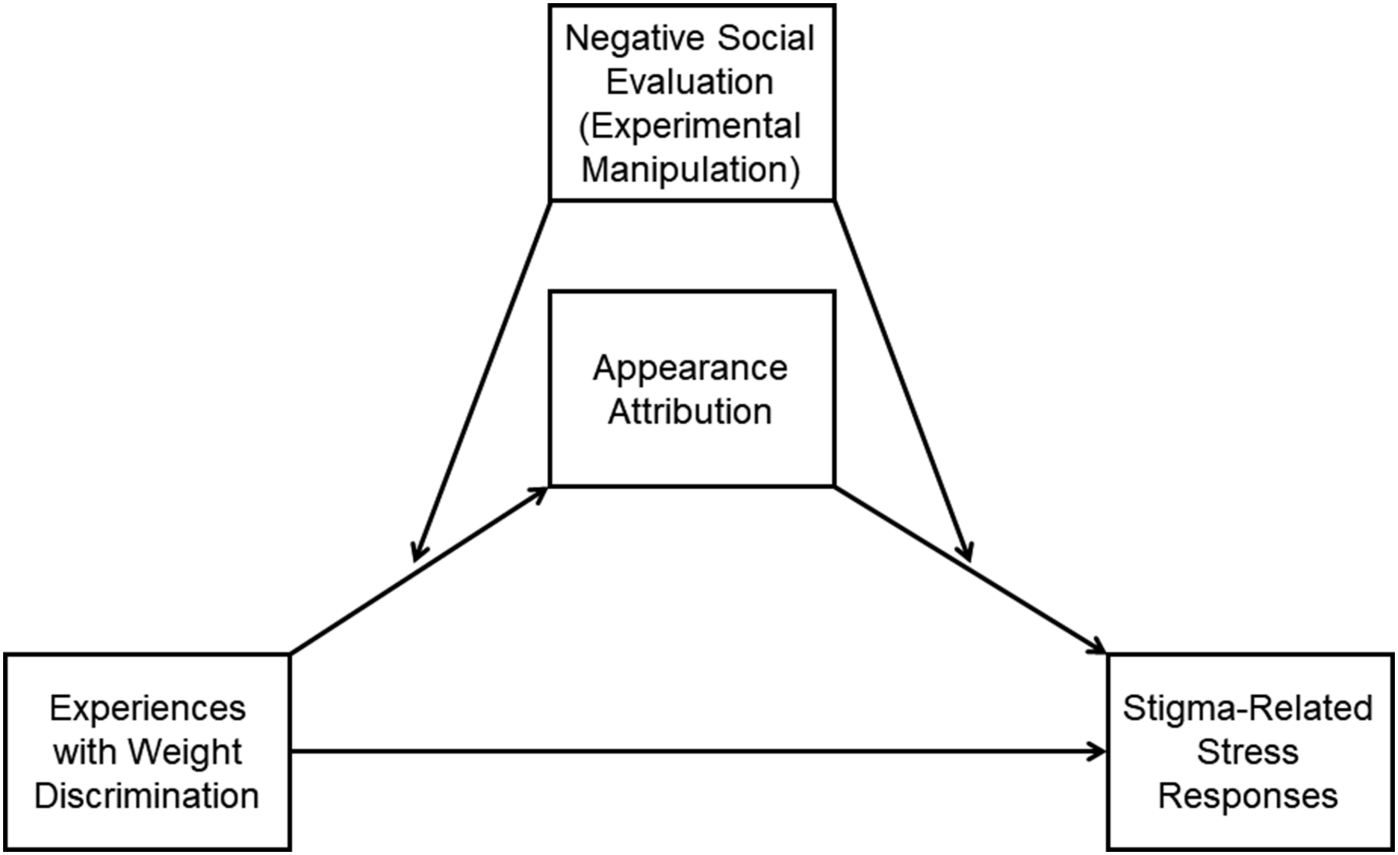
Conceptual model: Appearance attribution is proposed to explain the link between experiences with weight discrimination and stigma‐related stress responses to ambiguous negative social evaluation (as manipulated in the current experiment)

Moreover, with repeated exposure to day‐to‐day weight discrimination, individuals with obesity may become more likely to attribute ambiguous forms of mistreatment or social rejection to their body weight. Frequent experiences with weight‐based discrimination are likely to make the possibility of experiencing such discrimination particularly salient in the future.^36^ Thus, previous experiences with weight discrimination might increase the likelihood that one would come to anticipate weight stigma and to interpret negative social behaviours as constituting weight‐based discrimination. See Figure 1 for the conceptual model depicting this hypothesized relationship and the framework guiding the current research.

The present study used an experimental, lab‐based approach to investigate attribution as a psychological mechanism underlying people's responses to ambiguous forms of weight stigma. Adults with obesity were randomly assigned to receive a negative (versus mildly positive) social evaluation, yet the reason for the evaluation was left ambiguous. Participants were asked to rate the extent to which they attributed the evaluation to their physical appearance. Afterwards they completed several measures and tasks designed to assess psychological and physiological stress responses to weight stigma. These indicators are represented in conceptual models describing the proposed pathways through which weight stigma leads to poor health^9^, ^27^, ^28^ and have been evaluated in previous experimental studies on weight stigma.^21^, ^22^, ^25^, ^37^, ^38^ The study tested the following hypotheses: (1) people who have experienced weight discrimination more (vs. less) frequently in the past would be more likely to attribute the ambiguous negative social evaluation to their appearance; (2) attributing negative social evaluation to one's appearance would be associated with elevated negative affect, lower state self‐esteem, decrements in cognitive functioning and higher cortisol reactivity; and (3) appearance attribution would statistically mediate the relationship between experiences with weight discrimination and stress responses to negative evaluation.

## 2 METHODS

### 2.1 Participants

The study was advertised via Facebook and flyers posted in the local community from March through August 2019. Interested participants completed an online screening survey assessing eligibility criteria. Eligibility requirements included being 18–64 years old and having a BMI ≥ 30 kg/m^2^. Exclusion criteria included being pregnant or planning to become pregnant in the near future, being diagnosed with Cushing syndrome, or currently or recently taking steroid medication, as these factors can affect cortisol. Participants were not informed of inclusion/exclusion criteria and did not know the study involved weight or obesity. Eligible individuals were invited to schedule an appointment. Of 2,780 completed screening surveys, 2,359 did not meet eligibility criteria, 279 were eligible but never scheduled an appointment, 31 were scheduled but cancelled or failed to attend and 111 completed the study. Two participants were subsequently excluded because they did not meet BMI eligibility criteria, resulting in a final sample of 109.

A power analysis was conducted using effect sizes from Maner and colleagues,^39^ which used a similar manipulation of negative interpersonal feedback. Findings from those studies produced medium‐to‐large effect sizes. Using the smallest observed effect size for a two‐way interaction between manipulated interpersonal feedback and a measured variable (social anxiety; *r* = .34) required a total of 68 participants to achieve .90 power. Further, a post hoc power analysis based on the effect size of the key predicted interaction in the current study (experimental condition by appearance attribution; *r* = .27) indicated that power was adequate (observed power = .89) to detect the hypothesized pattern.

Sample characteristics are provided in Table 1. The sample was primarily female (81%) and included participants from a variety of ethnic (21% Latino) and racial backgrounds (67% White, 20% Black or African American). Average BMI was 38.43 kg/m^2^ (SD = 7.67).

**TABLE 1:** Sample characteristics by experimental condition (N = 109)

	Control (n = 54) N (%) M (SD)	Negative Feedback (n = 55) N (%) M (SD)
Age (years)	33.33 (12.91)	32.87 (13.75)
Gender		
Male	11 (20)	10 (18)
Female	43 (80)	45 (82)
Latino		
No	43 (80)	43 (78)
Yes	11 (20)	12 (22)
Race		
American Indian or Alaska Native	0 (0)	1 (2)
Asian	2 (4)	4 (7)
Black or African American	12 (22)	10 (18)
White	37 (69)	36 (66)
Multi‐racial	1 (2)	1 (2)
Unknown or missing	2 (4)	3 (6)
Marital status		
Never married	34 (63)	34 (62)
Married	15 (28)	15 (27)
Widowed/divorced/separated	5 (9)	6 (11)
Education		
High school diploma/GED	2 (4)	3 (6)
Some college	14 (26)	23 (42)
College graduate	21 (39)	14 (26)
Graduate/Professional degree	17 (32)	15 (27)
Annual family income		
≤$24,999	12 (22)	9 (16)
$25,000‐$49,999	12 (22)	15 (27)
$50,000‐$74,999	11 (20)	8 (15)
$75,000‐$99,999	8 (15)	5 (9)
$100,000‐$124,999	2 (4)	6 (11)
$125,000‐$149,999	1 (2)	3 (6)
≥$150,000	4 (7)	5 (9)
Do not want to answer	4 (7)	4 (7)
BMI (kg/m^2^)^a^	39.83 (8.48)	37.05 (6.56)
Perceived weight^b^	5.58 (0.89)	5.31 (0.98)
Salivary cortisol at baseline (nmol/L^2^)	3.34 (1.73)	3.14 (1.94)

*Note.* Percentages may not sum to 100 due to rounding error. *T* tests and chi‐square analyses were used to compare participants across conditions. No significant differences were observed.

^a^ Body mass index (BMI in kg/m^2^) was computed from measured height and weight. Fourteen participants declined to be weighed in the lab and instead provided self‐reported weight. For these participants, we used the average of the two self‐reported weights (screening survey and lab) when calculating BMI.

^b^ Perceived weight was assessed with the following item: “How would you rate your current body size?” (1 = very underweight to 7 = very overweight).

### 2.2 Procedure

The study took place in a laboratory on campus between 12:00 and 8:00 pm. Sessions lasted approximately 75 minutes and were led by two research assistants: a primary experimenter who assisted the participant and a secondary experimenter who ostensibly assisted the partner, but actually played the partner role during the first interaction. To disguise the purpose of the study, participants were told the study investigated how different modes of communication affect social interactions and health. Further, participants were told: “During today's study you will be interacting with a partner who is in the lab next door. Your first interaction will take place over instant messaging, where you will communicate by text only. Then you will interact by video voice mail, where you will each record and watch a brief video. And then finally, you will interact with your partner face‐to‐face. After each interaction you will be asked to provide impressions of your partner and your partner will do the same for you.”

After participants provided written informed consent and completed baseline measures, the experimenter collected a baseline saliva sample (approximately 15–20 minutes into the study). Next participants completed an ~ 4‐minute instant messaging interaction with their ostensible partner (the second experimenter) in which they exchanged basic information about themselves (eg, their first name and where they live). Participants provided feedback on the first interaction and then exchanged feedback with their partner. To support the cover story, the feedback included two items about instant messaging (“How effective do you think instant messaging is for communicating with your partner?” and “How easy or difficult was it to form an impression of your partner using instant messaging?”) and two items about their impression of their partner (“How much are you looking forward to the video voicemail interaction with your partner?” 1 = not at all to 7 = very much and “At this point, what is your overall impression of your partner?” 1 = very negative to 7 = very positive). All participants received relatively positive feedback after the first interaction (ratings of “6” for the two impression items).

Next participants recorded an ~ 3‐minute video to further introduce themselves to their partner. A webcam recorded the participant's face and entire upper body (so that their weight was readily apparent) while seated at the computer. Participants were given a list of “getting to know you” questions to facilitate creation of their video (eg, What pets did you have while you were growing up?). Participants then exchanged videos, watched their partner's video (a pre‐recorded video matched to participant gender) and provided feedback on their partner's video via ratings (similar to the first interaction) as well as written feedback. The secondary experimenter collected the participant's feedback and gave the participant their partner's ostensible feedback. This feedback constituted the experimental manipulation.

Participants were randomized using a 1:1 allocation ratio to receive either control or negative feedback. Participants in the control condition received mildly positive feedback similar to the first evaluation: scores of “6” on both impression items (where 7 = very positive) and the following comment, tailored to participant gender: “She seems nice and I liked her video.”). Participants in the negative feedback condition received scores of “2” on both impression items (where 1 = very negative) and the following comment: “Her video was fine, but I guess I'm not that excited about talking face‐to‐face.” Notably, no reason was provided for this relatively negative social evaluation.

After receiving this feedback, participants completed a survey assessing their attributions for the feedback and self‐reported outcome variables (emotions and self‐esteem). Next, participants performed a cognitive functioning task and completed additional survey questions. Participants provided a second saliva sample approximately 20 minutes after the experimental manipulation. Research suggests that it takes approximately 15 minutes for changes in cortisol concentrations to be detectable in saliva.^40^, ^41^ The experimenter then probed the participant for suspicion (eg, “Up through this point, has anything about the study struck you as strange or unusual?”) prior to conducting a detailed debriefing that explained the study purpose and clarified that the partner and feedback were fictitious. Lastly, participants completed a final survey that assessed previous experiences with weight discrimination, along with additional individual difference measures not reported in this paper. These measures were intentionally collected at the end of the study (versus earlier) to cloak the study's focus on weight stigma. It is important to note that self‐reported levels of previous weight discrimination did not vary by condition (control: M = 1.58, SD = 0.69; negative feedback: M = 1.63, SD = 0.84; *t*(106) = −0.31, *p* = .76), confirming that these reports were unaffected by the experimental manipulation. Before dismissal, the experimenter measured the participant's height and weight to confirm BMI. Participants were compensated $40. The primary experimenter remained blind to condition until all outcome variables were collected.

Two coders reviewed notes from the suspicion probe and determined that 10 participants (control: n = 3; negative feedback: n = 7) expressed suspicion about their partner and/or the veracity of the feedback received from the partner. Findings were nearly identical whether these participants are included or excluded; thus, all participants were retained for analyses.

### 2.3 Measures

#### 2.3.1 Sample characteristics

Participants reported demographic characteristics and self‐perceived weight (“How would you rate your current body size?” 1 = very underweight and 7 = very overweight). BMI (kg/m^2^) was computed from lab‐measured height and weight.

#### 2.3.2 Experiences with weight discrimination

Perceived experiences with weight discrimination were assessed with six items adapted from research by Williams and colleagues^42^ that have been used in previous weight stigma research.^13^ Participants were asked to rate the frequency with which they experience unfair treatment due to their weight on a day‐to‐day basis (eg, “You are treated with less courtesy or respect than other people because of your weight.” And “You receive poorer service than other people at restaurants or stores because of your weight.”). Items were accompanied by a 6‐point response scale (1 = never, 2 = less than once a year, 3 = a few times a year, 4 = a few times a month, 5 = at least once a week and 6 = almost every day). Items were averaged to create a composite score (*α* = .84) with higher scores representing greater perceived frequency of experiences with weight discrimination.

#### 2.3.3 Attributions

Attributions for partner feedback were modelled after items from Major and colleagues.^43^ Participants indicated the extent to which they thought their partner's feedback was due to “your appearance,” in addition to other plausible attributions (“your personality, your partner's personality, your gender, and your partner's gender”), and two distractor items included to support the cover story (eg, “your speaking style”). The phrase “your appearance” (rather than “your weight”) was used deliberately to mask the study's focus on weight stigma. Attributions were rated on a 5‐point scale (1 = not at all, 2 = a little bit, 3 = somewhat, 4 = very much and 5 = extremely) with higher scores indicating greater attribution to that domain.

#### 2.3.4 Negative affect

An adapted version of the Discrete Emotions Questionnaire (DEQ)^44^ was used to assess emotions after the experimental manipulation. Five emotions (anger, sadness, happiness, fear and anxiety) were assessed along with and an additional emotion relevant to weight stigma (ie, shame).^26^, ^45^ Each emotion was assessed with three to five adjectives. Participants indicated the extent to which they felt each emotion at that moment (1 = not at all, 2 = slightly, 3 = somewhat, 4 = moderately, 5 = quite a bit, 6 = very much and 7 = extremely). A composite score for each emotional state was computed by taking the average of the relevant adjectives. A total negative affect composite score was computed by averaging the anger, sadness, anxiety, fear and shame adjectives (*α* = .87). Higher scores indicate more emotion/negative affect.

#### 2.3.5 State self‐esteem

The state self‐esteem scale^46^ assessed social and appearance self‐esteem after the experimental manipulation. Social self‐esteem (eg, “I feel concerned about the impression I am making” [reverse scored]) and appearance self‐esteem (eg, “I am pleased with my appearance right now.”) were assessed with seven and six items, respectively, using a 5‐point scale (1 = not at all, 2 = a little bit, 3 = somewhat, 4 = very much and 5 = extremely). Items were averaged to create two composite scores (social self‐esteem *α* = .90; appearance self‐esteem *α* = .87) with higher scores representing higher state self‐esteem.

#### 2.3.6 Cognitive functioning

The Stroop task was used to assess cognitive functioning.^47^ The task is used widely for assessing people's ability to inhibit an automatic response (reading) in favour of performing a more controlled task (colour naming). Participants were presented with a series of cards. Each card (trial) contained 30 words (the names of various colours) arranged in rows. Reading from left to right, participants were instructed to name the ink colour of each word as quickly as possible while avoiding mistakes. On filler trials, the ink colour and word name were matched (eg, the word “red” was printed in red ink), so naming the colour was simple. On incongruent trials, however, the ink colour and word name were mismatched (eg, the word “red” was printed in blue ink). This required participants to suppress the automatic response of reading the word. The task thus measures cognitive control with longer reaction times indicating that a participant was less able to suppress the automatic response. Participants began with two abbreviated practice trials followed by 10 trials, three of which were filler (congruent) trials and seven of which were incongruent trials. The experimenter recorded response time in seconds for each trial. Cognitive functioning scores were calculated by computing the average time taken to complete incongruent trials. Higher response latencies reflected lower cognitive functioning. Scores were not calculated for two participants who failed to follow instructions. Two additional participants had very high response times (>3 SDs above the mean) and were excluded from analyses.

#### 2.3.7 Salivary cortisol

Participants provided saliva samples at baseline and 20 minutes after the experimental manipulation (at follow‐up) via passive drool. Within 2 hours of collection, saliva samples were frozen at −20°C. Samples were thawed, centrifuged at 2000 *g* for 10 minutes and analysed using a competitive solid phase time‐resolved fluorescence immunoassay with flouromeric end point detection (dissociation‐enhanced lanthanide fluorescence immunoassay). Samples were analysed in duplicate (average coefficient of variation across samples = 5.16%). Participants were instructed to refrain from eating, drinking any beverages besides water, smoking or exercising within the 2‐hour period before their session. Five participants reported eating, drinking and/or smoking prior to the session; however, findings remained the same whether these participants were included/excluded so they are included for all analyses. One participant with a very high follow‐up cortisol value (>3 SDs above the mean) was excluded from analysis. Baseline and follow‐up cortisol values were not skewed (skewness values were below 1.0); thus, variables were not subjected to a log transformation before analysis.

### 2.4 Statistical analysis

Analyses were performed using IBM SPSS Statistics 26 and the PROCESS macro.^48^ To assess whether randomization was successful, *t* tests and chi‐square analyses were used to compare baseline sample characteristics across the two conditions. Ordinary least squares regression analysis was used to examine whether experimental condition interacted with experiences with weight discrimination to affect appearance attribution. To evaluate the form of the interaction, the effect of weight discrimination on appearance attribution was examined for participants in the control vs. negative feedback condition. All predictor variables were centred prior to analysis. Bootstrap confidence intervals were estimated using 5,000 samples. Following the same procedure, a series of ordinary least squares regression analyses were conducted to examine whether appearance attribution interacted with experimental condition to affect primary outcome measures (negative affect along with all six individual emotions, social and appearance self‐esteem, cognitive functioning and salivary cortisol at follow‐up). Baseline cortisol was included as a covariate in the analysis predicting cortisol at follow‐up. In addition, a repeated measures analysis of variance was conducted to assess effects of experimental condition, appearance attribution and their interaction on cortisol reactivity (change in cortisol from baseline to follow‐up). Conditional process analysis^48^ was used to examine whether appearance attribution statistically mediated the link between experiences with weight discrimination and stigma‐related stress responses to the negative feedback manipulation (“moderated mediation” Model 58 in PROCESS). All analyses were then repeated while controlling for (1) BMI and (2) perceived weight. The University Human Subjects Committee approved all study procedures.

## 3 RESULTS

Sample characteristics are provided in Table 1. Participants were randomly assigned to receive control (n = 54) or negative feedback (n = 55). Sample characteristics did not differ by experimental condition, indicating successful randomization. Descriptive statistics for primary outcomes by experimental condition are provided in Table 2.

**TABLE 2:** Descriptive statistics for primary outcome variables among participants in the control condition and negative feedback condition (N = 109)

	Control (n = 54) M (SD)	Negative Feedback (n = 55) M (SD)
Negative affect	1.32 (0.35)	2.05 (1.12)
Social self‐esteem	3.79 (0.88)	3.56 (1.05)
Appearance self‐esteem	2.91 (0.84)	2.77 (0.95)
Cognitive functioning	24.48 (4.80)	25.45 (5.59)
Salivary cortisol at follow‐up (nmol/L^2^)	2.74 (1.80)	2.33 (1.49)

*Note.* Negative affect was assessed as the extent to which participants were experiencing five negative emotions (anger, sadness, fear, anxiety and shame) after receiving the experimental manipulation. Each emotion was assessed with multiple adjectives rated on a 7‐point scale (1 = not at all to 7 = extremely). Adjectives were combined to create a single composite with higher scores representing more negative affect. Social and appearance self‐esteem were each assessed on a 5‐point scale (1 = not at all to 5 = extremely) with higher scores representing higher state self‐esteem. Cognitive functioning scores were calculated by computing the average time in seconds taken to complete the incongruent trials. Higher scores (ie, longer response times) indicate worse performance.

Results from the analysis predicting appearance attribution from experimental condition, experiences with weight discrimination and their interaction are presented in Table 3. Experiences with weight discrimination and experimental condition interacted to predict appearance attribution. When participants received negative feedback, there was a positive relationship between weight discrimination and appearance attribution such that participants who experienced more frequent weight discrimination were more likely to attribute the (negative) feedback to their appearance. In contrast, when participants received control feedback, there was a negative relationship between weight discrimination and appearance attribution, such that participants who experienced more frequent weight discrimination were less likely to attribute the (positive) feedback to their appearance.

**TABLE 3:** Results from the regression analysis predicting appearance attribution from experimental condition, experiences with weight discrimination and their interaction

	*B*	SE	*t*	*p*	95% CI
Condition	0.53	0.21	2.52	.01	0.11, 0.94
Weight discrimination	0.01	0.14	0.09	.93	−0.26, 0.29
Condition × weight discrimination	0.91	0.28	3.24	<.01	0.35, 1.46
Control condition^a^	−0.45	0.22	−2.06	.04	−0.88, −0.02
Negative feedback condition^a^	0.46	0.18	2.60	.01	0.11, 0.80

*Note.* Condition: 1 = control; 2 = negative feedback; *B* = unstandardized regression coefficient; SE = standard error; 95% CI = bootstrap 95% confidence interval.

^a^ Interactions were interpreted by examining the conditional effect of weight discrimination on appearance attribution for participants in the control vs. negative feedback condition.

Results from analyses predicting primary outcome variables from experimental condition, appearance attribution and their interaction are presented in Table 4. As predicted, an interaction between experimental condition and appearance attribution was observed for negative affect, appearance self‐esteem and cognitive functioning. Examining the form of interaction indicated that appearance attribution was not associated with negative affect, appearance self‐esteem or cognitive functioning among participants who received control feedback. However, among participants who received negative feedback, those with higher appearance attribution scores reported more negative affect and lower appearance self‐esteem and performed worse on the cognitive task. It should be noted in the analysis predicting appearance self‐esteem that both the overall interaction effect (*p* = .06) and conditional effect in the negative feedback condition (*p* = .06) only approached statistical significance. Findings for the individual emotions of anger, sadness, fear and shame mirrored the interactive pattern observed for negative affect; however, only main effects of condition were observed for happiness (more happiness in the control vs. negative feedback condition) and anxiety (more anxiety in the negative feedback vs. control condition; data not shown). No effects of experimental condition or appearance attribution were observed for social self‐esteem or cortisol at follow‐up (controlling for baseline cortisol; see Table 4). Similarly, a repeated measures analysis of variance indicated that cortisol values did not change from baseline to follow‐up and neither the main effects of condition or appearance attribution nor the interaction between those variables were statistically significant (results not shown).

**TABLE 4:** Results from regression analyses predicting primary outcome variables from experimental condition, appearance attribution and their interaction

	*B*	SE	*t*	*p*	95% CI
Negative affect					
Condition	0.62	0.15	4.06	<.01	0.32, 0.93
Appearance attribution	0.19	0.07	2.91	.01	0.06, 0.33
Condition × appearance attribution	0.40	0.13	2.98	<.01	0.13, 0.66
Control condition^a^	0.00	0.10	−0.69	.95	−0.20, 0.18
Negative feedback condition^a^	0.39	0.09	4.21	<.01	0.21, 0.58
Social self‐esteem					
Condition	−0.17	0.19	−0.89	.38	−0.55, 0.21
Appearance attribution	−0.12	0.08	−1.49	.14	−0.29, 0.04
Condition × appearance attribution	−0.11	0.17	−0.65	.52	−0.44, 0.22
Control condition^a^	—	—	—	—	—
Negative feedback condition^a^	—	—	—	—	—
Appearance self‐esteem					
Condition	−0.11	0.18	−0.63	.54	−0.46, 0.24
Appearance attribution	−0.06	0.08	−0.73	.47	−0.21, 0.10
Condition × appearance attribution	−0.30	0.15	−1.94	.06	−0.60, 0.01
Control condition^a^	0.09	0.11	0.86	.39	−0.12, 0.31
Negative feedback condition^a^	−0.20	0.11	−1.91	.06	−0.41, 0.01
Cognitive functioning					
Condition	0.35	1.00	0.34	.73	−1.64, 2.33
Appearance attribution	1.06	0.44	2.42	.02	0.19, 1.93
Condition × appearance attribution	2.11	0.88	2.40	.02	0.37, 3.85
Control condition^a^	0.04	0.61	0.06	.95	−1.18, 1.26
Negative feedback condition^a^	2.15	0.63	3.42	<.01	0.90, 3.39
Salivary cortisol at follow‐up					
Condition	−0.30	0.29	−1.04	.30	−0.88, 0.27
Appearance attribution	−0.02	0.13	−0.16	.87	−0.27, 0.23
Salivary cortisol at baseline^b^	0.48	0.08	6.11	<.01	0.33, 0.64
Condition × appearance attribution	0.00	0.25	−0.00	1.00	−0.50, 0.50
Control condition^a^	—	—	—	—	—
Negative feedback condition^a^	—	—	—	—	—

*Note.* Condition: 1 = control; 2 = negative feedback; *B* = unstandardized regression coefficient; SE = standard error; 95% CI = bootstrap 95% confidence interval.

^a^ Interactions were interpreted by examining the conditional effect of appearance attribution on the outcome variable for participants in the control vs. negative feedback condition. Conditional effects are generated by PROCESS only for those interactions that reach marginal statistical significance or better (*p* < .10). Thus, conditional effects are not reported for those outcome variables for which no interaction was observed.

^b^ Analysis controlled for baseline salivary cortisol.

Next, the complete moderated mediation model (Figure 1) was estimated for negative affect, appearance self‐esteem and cognitive functioning (those outcomes for which an experimental condition by appearance attribution interaction was observed). Evidence in support of moderated mediation was observed for negative affect (index of moderated mediation = 0.17, bootstrap standard error [SE] = 0.10, and bootstrap 95% confidence interval [CI] = 0.01, 0.39) and cognitive functioning (index of moderated mediation = 1.14, bootstrap SE = 0.66, and bootstrap 95% CI = 0.003, 2.55). The bootstrap confidence interval for the “index of moderated mediation” did not contain zero for either of these outcome variables, thus providing support for moderated mediation. Participants who experienced more frequent weight discrimination in the past were more likely to attribute the negative (vs. control) feedback to their appearance. In turn, participants who were more likely to attribute the negative (vs. control) feedback to their appearance reported more negative affect and demonstrated poorer cognitive functioning. And, finally, attributing the negative feedback to their weight statistically explained the link between past experiences with weight discrimination and the outcome variables (negative affect and cognitive functioning). Neither model provided support for a direct effect of weight discrimination on negative affect or cognitive functioning. No support for moderated mediation was found for appearance self‐esteem (index of moderated mediation = −0.04, bootstrap SE = 0.07, bootstrap 95% CI = −0.18, 0.11); however, a direct effect of experiences with weight discrimination was observed such that participants who had experienced more frequent weight discrimination reported lower appearance self‐esteem (unstandardized regression coefficient = −0.40, SE = 0.11, *p* = .01).

All primary analyses were repeated while controlling for (a) BMI and then (b) perceived weight. Results were essentially identical with one exception: When controlling for BMI in the moderated mediation analysis predicting cognitive functioning, the confidence interval for the index of moderated mediation contained zero. All other effects remained (data not shown).

Finally, ancillary analyses were conducted to assess whether the observed effects were specific to appearance attribution. Means and standard deviations for all attribution measures are provided in Table 5. Relative to participants in the negative feedback condition, participants in the control condition were more likely to attribute the partner feedback to their personality, their gender and their partner's gender. The only attribution for which a significant difference in the opposite direction was observed was appearance attribution. Participants in the negative feedback condition were more likely to attribute the partner feedback to their appearance than were participants in the control feedback condition.

**TABLE 5:** Descriptive statistics for attributions for partner feedback by experimental condition

Attribution domain	Control (n = 54) M (SD)	Negative Feedback (n = 55) M (SD)
Participant personality attribution	3.87 (0.83)	2.96 (1.14)^**^
Partner personality attribution	3.54 (0.79)	3.36 (1.10)
Participant gender attribution	2.11 (1.27)	1.48 (0.93)^**^
Partner gender attribution	2.20 (1.28)	1.42 (0.85)^**^
Participant appearance attribution	2.50 (1.11)	3.04 (1.14)^*^

*Note.* Attributions were rated on a 5‐point scale (1 = not at all, 2 = a little bit, 3 = somewhat, 4 = very much and 5 = extremely) with higher scores indicating more attribution to that domain. For example, participant personality attribution was assessed with the following question: “To what extent do you think your partner's feedback of you from the second (video) interaction was due to your personality?” *T* tests were conducted to compare attribution ratings for participants in the control vs. negative feedback condition.

^*^ *p* < .05.

^**^ *p* < .01.

## 4 DISCUSSION

The present study illustrates the important role of attribution in people's responses to weight stigma. In a carefully controlled laboratory experiment, participants with obesity who reported experiencing more frequent weight discrimination in their day‐to‐day lives were significantly more likely to attribute negative but ambiguous social feedback to their physical appearance. Moreover, participants who attributed the negative feedback to their appearance in turn displayed heightened negative affect, lower appearance self‐esteem and poorer cognitive functioning. Findings suggest that attributions may serve as an important early‐stage psychological process underlying immediate psychological and cognitive responses to ambiguous forms of weight stigma.

Most models of social perception assume that when people perceive the behaviour of others, those perceptions reflect both features of the social situation and the subjective interpretation of the individual.^29^, ^30^ Consistent with this view, individual differences in people's perceptions of weight discrimination could reflect features of the situation (ie, the frequency and intensity with which people are subjected to unfair treatment based on their weight) and subjective interpretations of other people's behaviour, such as attributing hostile but ambiguous behaviours to one's weight. Ultimately, both features of the situation and one's subjective interpretation of others' behaviour are likely influenced by a range of factors. The current study confirms that such interpretations – and attributional processes in particular – may play a key role in the downstream effects of perceived weight discrimination on health.

Findings from the present study fit with theories that distinguish between people's experiences with unfair treatment (also referred to as “enacted stigma”) and the tendency to internalize the negative stereotypes and attitudes that society holds toward a socially devalued trait (also referred to as “felt” or “self‐directed” stigma).^5^, ^8^, ^10^, ^49^ People who have internalized weight stigma tend to evaluate themselves negatively for their weight and anticipate that others will stigmatize them for their weight.^8^, ^10^ Internalized weight stigma may play an especially important role in guiding the interpretation of negative social encounters when the perpetrator's motives are ambiguous. In such circumstances, people's expectations regarding mistreatment may shape their attributions, leading them to interpret negative social behaviours as reflecting weight‐based discrimination. Indeed, weight was never mentioned in the current study: participants in the experimental condition received a negative evaluation from their partner, yet the reason for the evaluation was not specified. Whether participants attributed the negative evaluation to their weight depended on their previous experiences with weight discrimination. Participants who perceived themselves as having been exposed more frequently to weight discrimination in the past were more likely to attribute the negative evaluation to their appearance. Such perceptions likely reflect a blend of people's previous experiences with weight discrimination as well as their level of internalized weight stigma.^5^, ^9^, ^10^

The current research also extends the literature by focusing directly on individual differences in responses to weight stigma among people with obesity. Previous experimental studies on weight stigma have tended to recruit participants with a range of BMIs (eg, from “average weight” to “obese”) and focus on comparing people who perceive themselves to be overweight (or have a BMI indicating “overweight”) to people who perceive themselves to be of normal weight (or have a BMI indicating “normal weight”).^21^, ^22^, ^23^, ^37^, ^50^ Unlike these studies, the present study was purposely limited to people who met criteria for obesity (BMI ≥ 30 kg/m^2^) and thus may be at heightened risk for experiencing weight‐based discrimination. This strategy allowed for an examination of individual differences that may shape people's responses to weight stigma by focusing exclusively on individual variability in responses to negative social evaluation among a sample of adults with obesity. This work thus has important implications for identifying and understanding factors that confer risk versus resilience to the negative effects of weight stigma in people with obesity.

The psychological responses observed among participants who attributed the negative social evaluation to their appearance (ie, increased negative affect, reduced cognitive functioning and appearance self‐esteem) could explain some of the downstream negative health consequences of weight stigma reported in the literature.^9^, ^11^, ^12^ High levels of negative affect, for example, could contribute to the development of anxiety and depression both of which are elevated in people subjected to weight discrimination.^51^ Similarly, lower levels of cognitive functioning could play a role in the maintenance of obesity, as cognitive functioning has been implicated in the regulation of eating behaviour.^27^ Impaired cognitive functioning in response to weight stigma could reflect distraction, consistent with evidence that rumination is a common emotion regulation response that can be triggered by exposure to discrimination.^52^, ^53^ More broadly, this research complements the existing epidemiological literature and provides new evidence for more proximal psychological processes through which weight discrimination may harm health.

Unlike previous studies,^21^, ^25^ no effects on cortisol reactivity were observed. Null findings may have been a function of study design features such as the wide range of study times during which cortisol was assessed (between 12‐8 pm), the absence of a recovery saliva sample and variability in participant age. In addition, it is possible that the negative feedback manipulation was not powerful enough to elicit change in stress hormones. Further research is needed to better understand individual variability in physiological reactions to ambiguous instances of weight discrimination using additional biomarkers of stress (eg, alpha‐amylase and pro‐inflammatory cytokines) beyond cortisol reactivity.^54^

The current study has several limitations. Participants were asked to rate the extent to which they attributed the feedback to their appearance rather than their weight. That appearance attributions were pronounced among participants who reported more frequent past experiences with weight discrimination suggests that participants were, in fact, thinking about their weight. Nevertheless, it is possible that participants may also have been considering other aspects of their physical appearance (eg, attractiveness and skin colour). Another limitation is that only one form of negative social evaluation (ie, a negative first impression among unacquainted individuals in a laboratory setting) was used. Future work should evaluate the extent to which the current findings generalize to other social contexts. Additionally, although weight discrimination tends to be more prevalent among women,^2^, ^4^, ^37^ the sample included a relatively small proportion of male participants. As research has documented the negative effects of weight discrimination for men's health,^55^ it is important that men are well represented in future research. Finally, although findings may have implications for the downstream effects of weight stigma, such long‐term outcomes were not examined in the present study. Future research would benefit from investigating the role of weight‐related attribution in contributing to poor mental and physical health outcomes associated with weight stigma.

Despite these limitations, this study is one of the first to identify an early‐stage psychological process underlying people's immediate responses to ambiguous weight stigma. Attributing signs of social bias or mistreatment to one's body weight could promote a cascade of responses that ultimately lead to poorer physical and psychological health. As such, attributional processes may be a key psychological factor that confers risk for or protection from the negative effects of weight‐based discrimination and may serve as a useful target for future intervention efforts.

## FUNDING

This research was funded in part by the Florida State University Council on Research and Creativity.

## CONFLICT OF INTEREST STATEMENT

The authors declared no conflict of interest.

## References

[1] Puhl RM , Heuer CA . The stigma of obesity: a review and update. Obesity. 2009;17:941‐964. 19165161 10.1038/oby.2008.636

[2] Dutton GR , Lewis TT , Durant N , et al. Perceived weight discrimination in the CARDIA study: differences by race, sex, and weight status. Obesity. 2014;22:530‐536. 23512948 10.1002/oby.20438 PMC3695009

[3] Himmelstein MS , Puhl RM , Quinn DM . Intersectionality: an understudied framework for addressing weight stigma. Am J Prev Med. 2017;53:421‐431. 28579331 10.1016/j.amepre.2017.04.003

[4] Spahlholz J , Baer N , Konig HH , Riedel‐Heller SG , Luck‐Sikorski C . Obesity and discrimination—a systematic review and meta‐analysis of observational studies. Obes Rev. 2016;17:43‐55. 26596238 10.1111/obr.12343

[5] Pearl RL . Weight bias and stigma: public health implications and structural solutions. Soc Issues Policy Rev. 2018;12:146‐182.

[6] Phelan SM , Burgess DJ , Yeazel MW , Hellerstedt WL , Griffin JM , van Ryn M . Impact of weight bias and stigma on quality of care and outcomes for patients with obesity. Obes Rev. 2015;16:319‐326. 25752756 10.1111/obr.12266 PMC4381543

[7] Lewis S , Thomas SL , Blood RW , Castle DJ , Hyde J , Komesaroff PA . How do obese individuals perceive and respond to the different types of obesity stigma that they encounter in their daily lives? A qualitative study. Soc Sci Med. 2011;73:1349‐1356. 21944718 10.1016/j.socscimed.2011.08.021

[8] Lillis J , Luoma JB , Levin ME , Hayes SC . Measuring weight self‐stigma: the weight self‐stigma questionnaire. Obesity. 2010;18:971‐976. 19834462 10.1038/oby.2009.353

[9] Major B , Tomiyama AJ , Hunger J . The negative and bidirectional effects of weight stigma on health In: Major B, Dovidio JF, Link BG, eds. The Oxford Handbook of Stigma, Discrimination, and Health. New York: Oxford University Press; 2018:499‐520.

[10] Pearl RL , Puhl RM . Weight bias internalization and health: a systematic review. Obes Rev. 2018;19:1141‐1163. 29788533 10.1111/obr.12701 PMC6103811

[11] Puhl RM , Heuer CA . Obesity stigma: important considerations for public health. Am J Public Health. 2010;100:1019‐1028. 20075322 10.2105/AJPH.2009.159491 PMC2866597

[12] Wu YK , Berry DC . Impact of weight stigma on physiological and psychological health outcomes for overweight and obese adults: a systematic review. J Adv Nurs. 2018;74:1030‐1042. 29171076 10.1111/jan.13511

[13] Hunger JM , Major B . Weight stigma mediates the association between BMI and self‐reported health. Health Psychol. 2015;34:172‐175. 25133837 10.1037/hea0000106 PMC4308542

[14] Potter L , Wallston K , Trief P , Ulbrecht J , Juth V , Smyth J . Attributing discrimination to weight: associations with well‐being, self‐care, and disease status in patients with type 2 diabetes mellitus. J Behav Med. 2015;38:863‐875. 26133488 10.1007/s10865-015-9655-0 PMC4628883

[15] Sutin AR , Stephan Y , Gerend MA , Robinson E , Daly M , Terracciano A . Perceived weight discrimination and performance in five domains of cognitive function. J Psychosom Res. 2020;131:109793 10.1016/j.jpsychores.2019.109793 31439334 PMC7002199

[16] Sutin AR , Stephan Y , Robinson E , Daly M , Terracciano A . Perceived weight discrimination and risk of incident dementia. Int J Obes (Lond). 2019;43:1130‐1134. 30250239 10.1038/s41366-018-0211-1 PMC6431581

[17] Sutin AR , Stephan Y , Terracciano A . Weight discrimination and risk of mortality. Psychol Sci. 2015;26:1803‐1811. 26420442 10.1177/0956797615601103 PMC4636946

[18] Jackson SE , Beeken RJ , Wardle J . Perceived weight discrimination and changes in weight, waist circumference, and weight status. Obesity. 2014;22:2485‐2488. 25212272 10.1002/oby.20891 PMC4236245

[19] Sutin AR , Terracciano A . Perceived weight discrimination and obesity. Plos ONE. 2013;8:e70048 10.1371/journal.pone.0070048 23894586 PMC3722198

[20] Tomiyama AJ , Carr D , Granberg EM , et al. How and why weight stigma drives the obesity ‘epidemic’ and harms health. BMC Med. 2018;16:123 10.1186/s12916-018-1116-5 30107800 PMC6092785

[21] Himmelstein MS , Incollingo Belsky AC , Tomiyama AJ . The weight of stigma: cortisol reactivity to manipulated weight stigma. Obesity. 2015;23:368‐374. 25522347 10.1002/oby.20959

[22] Major B , Eliezer D , Rieck H . The psychological weight of weight stigma. Soc Psychol Personal Sci. 2012;3:651‐658.

[23] Major B , Hunger JM , Bunyan DP , Miller CT . The ironic effects of weight stigma. J Exp Soc Psychol. 2014;51:74‐80.

[24] Pascoe EA , Richman LS . Perceived discrimination and health: a meta‐analytic review. Psychol Bull. 2009;135:531‐554. 19586161 10.1037/a0016059 PMC2747726

[25] Schvey NA , Puhl RM , Brownell KD . The stress of stigma: exploring the effect of weight stigma on cortisol reactivity. Psychosom Med. 2014;76:156‐162. 24434951 10.1097/PSY.0000000000000031

[26] Tomiyama AJ . Weight stigma is stressful. a review of evidence for the cyclic obesity/weight‐based stigma model. Appetite. 2014;82:8‐15. 24997407 10.1016/j.appet.2014.06.108

[27] Tomiyama AJ . Stress and obesity. Annu Rev Psychol. 2019;70:703‐718. 29927688 10.1146/annurev-psych-010418-102936

[28] Tomiyama AJ , Epel ES , McClatchey TM , et al. Associations of weight stigma with cortisol and oxidative stress independent of adiposity. Health Psychol. 2014;33:862‐867. 25068456 10.1037/hea0000107 PMC4677673

[29] Kruglanski AW . Lay epistemics and human knowledge. New York: Plenum Press; 1989.

[30] McArthur LZ , Baron RM . Toward an ecological theory of social perception. Psychol Rev. 1983;90:215‐238.

[31] Kelley HH . Attribution theory in social psychology In: Levine D, ed. Nebraska Symposium on Motivation. Lincoln: University of Nebraska Press; 1967:192‐238.

[32] Kunda Z . The case for motivated reasoning. Psychol Bull. 1990;108:480‐498. 2270237 10.1037/0033-2909.108.3.480

[33] Weiner B . An attributional theory of achievement motivation and emotion. Psychol Rev. 1985;92:548‐573. 3903815

[34] Lazarus RS . From psychological stress to the emotions: a history of changing outlooks. Annu Rev Psychol. 1993;44:1‐21. 8434890 10.1146/annurev.ps.44.020193.000245

[35] Lazarus RS , Folkman S . Stress, appraisal, and coping. New York: Springer; 1984.

[36] Vartanian LR , Pinkus RT , Smyth JM . The phenonmenology of weight stigma in everyday life. J Contextual Behav Sci. 2014;3:196‐202.

[37] Blodorn A , Major B , Hunger J , Miller C . Unpacking the psychological weight of weight stigma: a rejection‐expectation pathway. J Exp Soc Psychol. 2016;63:69‐76. 26752792 10.1016/j.jesp.2015.12.003 PMC4702265

[38] Hunger JM , Blodorn A , Miller CT , Major B . The psychological and physiological effects of interacting with an anti‐fat peer. Body Image. 2018;27:148‐155. 30267954 10.1016/j.bodyim.2018.09.002

[39] Maner JK , DeWall CN , Baumeister RF , Schaller M . Does social exclusion motivate interpersonal reconnection? Resolving the “porcupine problem”. J Pers Soc Psychol. 2007;92:42‐55. 17201541 10.1037/0022-3514.92.1.42

[40] Maner JK , Miller SL , Schmidt NB , Eckel LA . The endocrinology of exclusion: rejection elicits motivationally tuned changes in progesterone. Psychol Sci. 2010;21:581‐588. 20424105 10.1177/0956797610362676

[41] Reschke‐Hernandez AE , Okerstrom KL , Bowles Edwards A , Tranel D . Sex and stress: men and women show different cortisol responses to psychological stress induced by the Trier social stress test and the Iowa singing social stress test. J Neurosci Res. 2017;95:106‐114. 27870432 10.1002/jnr.23851 PMC5120613

[42] Williams DR , Yan Y , Jackson JS , Anderson NB . Racial differences in physical and mental health: socio‐economic status, stress and discrimination. J Health Psychol. 1997;2:335‐351. 22013026 10.1177/135910539700200305

[43] Major B , Quinton WJ , Schmader T . Attributions to discrimination and self‐esteem: impact of group identification and situational ambiguity. J Exp Soc Psychol. 2003;39:220‐231.

[44] Harmon‐Jones C , Bastian B , Harmon‐Jones E . The discrete emotions questionnaire: a new tool for measuring state self‐reported emotions. Plos ONE. 2016;11:e0159915 https://10.1371/journal.pone.0159915 27500829 10.1371/journal.pone.0159915 PMC4976910

[45] Dickerson SS , Gruenewald TL , Kemeny ME . When the social self is threatened: shame, physiology, and health. J Pers. 2004;72:1191‐1216. 15509281 10.1111/j.1467-6494.2004.00295.x

[46] Heatherton TF , Polivy J . Development and validation of a scale for measuring state self‐esteem. J Pers Soc Psychol. 1991;60:895‐910.

[47] MacLeod CM . Half a century of research on the Stroop effect: an integrative review. Psychol Bull. 1991;109:163‐203. 2034749 10.1037/0033-2909.109.2.163

[48] Hayes AF . Introduction to mediation, moderation, and conditional process analysis: a regression‐based approach. 2nd ed. New York: The Guilford Press; 2018.

[49] Corrigan PW , Larson JE , Rusch N . Self‐stigma and the “why try” effect: impact on life goals and evidence‐based practices. World Psychiatry. 2009;8:75‐81. 19516923 10.1002/j.2051-5545.2009.tb00218.x PMC2694098

[50] Crocker J , Cornwell B , Major B . The stigma of overweight: affective consequences of attributional ambiguity. J Pers Soc Psychol. 1993;64:60‐70. 8421252 10.1037//0022-3514.64.1.60

[51] Hatzenbuehler ML , Keyes KM , Hasin DS . Associations between perceived weight discrimination and the prevalence of psychiatric disorders in the general population. Obesity. 2009;17:2033‐2039. 19390520 10.1038/oby.2009.131 PMC3767420

[52] Hatzenbuehler ML . How does sexual minority stigma “get under the skin”? A psychological mediation framework. Psychol Bull. 2009;135:707‐730. 19702379 10.1037/a0016441 PMC2789474

[53] Hatzenbuehler ML , Nolen‐Hoeksema S , Dovidio J . How does stigma “get under the skin”?: the mediating role of emotion regulation. Psychol Sci. 2009;20:1282‐1289. 19765237 10.1111/j.1467-9280.2009.02441.x PMC3687354

[54] Nater UM , Skoluda N , Strahler J . Biomarkers of stress in behavioural medicine. Curr Opin Psychiatry. 2013;26:440‐445. 23867656 10.1097/YCO.0b013e328363b4ed

[55] Himmelstein MS , Puhl RM , Quinn DM . Overlooked and understudied: health consequences of weight stigma in men. Obesity. 2019;27:1598‐1605. 31364819 10.1002/oby.22599

